# Nouns, verbs, objects, actions, and abstractions: Local fMRI activity indexes semantics, not lexical categories

**DOI:** 10.1016/j.bandl.2014.03.001

**Published:** 2014-05

**Authors:** Rachel L. Moseley, Friedemann Pulvermüller

**Affiliations:** a MRC Cognition and Brain Sciences, Unit, 15 Chaucer Road, CB2 7EF, Cambridge, UK; b Autism Research Centre, Department of Psychiatry, University of Cambridge, Douglas House, 18b Trumpington Road, CB2 8AH, Cambridge, UK; c Brain Language Laboratory, Department of Philosophy and Humanities, Freie Universität Berlin, Habelschwerdter Allee 45, 14195 Berlin, Germany

**Keywords:** Embodied meaning, fMRI, Grammatical category, Lexical category, Semantics

## Abstract

Noun/verb dissociations in the literature defy interpretation due to the confound between lexical category and semantic meaning; nouns and verbs typically describe concrete objects and actions. Abstract words, pertaining to neither, are a critical test case: dissociations along lexical-grammatical lines would support models purporting lexical category as the principle governing brain organisation, whilst semantic models predict dissociation between concrete words but not abstract items. During fMRI scanning, participants read orthogonalised word categories of nouns and verbs, with or without concrete, sensorimotor meaning. Analysis of inferior frontal/insula, precentral and central areas revealed an interaction between lexical class and semantic factors with clear category differences between concrete nouns and verbs but not abstract ones. Though the brain stores the combinatorial and lexical-grammatical properties of words, our data show that topographical differences in brain activation, especially in the motor system and inferior frontal cortex, are driven by semantics and not by lexical class.

## Highlights

• Concrete nouns and verbs elicit different brain signatures in frontocentral cortex.
• Abstract nouns and verbs fail to elicit different brain activation patterns.
• Concrete verbs activate motor and premotor cortex more strongly than concrete nouns.
• Concrete nouns activate inferior frontal areas more strongly than concrete verbs.
• Lexical category distinctions in middle temporal cortex cannot be confirmed.

## 1 Introduction

The neurobiological basis of noun and verb processing has been elucidated by cognitive neuroscience research. A range of neuropsychological (Damasio & Tranel, 1993; Daniele, Giustolisi, Silveri, Colosimo, & Gainotti 1994; Kemmerer, Rudrauf, Manzel, & Tranel 2012; Miceli, Silveri, Villa, & Caramazza 1984; Neininger & Pulvermueller, 2001; Neininger & Pulvermüller, 2003) and brain imaging studies (Bedny, Caramazza, Grossman, Pascual-Leone, & Saxe 2008; Perani et al. 1999; Price, Wise, & Frackowiak 1996; Pulvermüller, Lutzenberger, & Preissl 1999) have linked these word classes to specific parts of the human brain, with verb processing associated with inferior frontal and middle temporal cortex and noun processing related to other temporal and parietal areas. Differences in grammatical or lexical class may not, however, be the principle factor in the neural differentiation between nouns and verbs. As one variable of interest, word meaning, or semantics, has frequently been discussed as an underlying determinant of noun/verb dissociations (Pulvermüller, Lutzenberger et al. 1999; Shallice, 1988; Vigliocco, Vinson, Druks, Barber, & Cappa 2011; Warrington & Shallice, 1984). An essential confound exists in the literature as most verbs are undeniably words used to speak about actions whereas most nouns refer to objects, so it is hardly possible to match and control for relevant semantic differences between the lexical classes; furthermore, were one to succeed in precisely matching sets of nouns and verbs for factors such as the concreteness of their object reference and intensity of their action relationship, one might, from a linguistic perspective, still argue that such selections would certainly be far from representing typical specimens from the lexical groups. Given this seemingly hopeless confound of lexical class with semantics, it is therefore unsurprising that many scholars have tried to trace the “lexical” differences to their semantic origins, at least as far as putative word class specific brain activation patterns are concerned.

Ingenious attempts have been made to clarify this issue by varying semantic properties within the lexical classes so that consistent noun/verb differences in brain activation – for example in the middle-temporal cortex (Bedny et al. 2008) – reveal more genuine lexical class differences. In addition, many authors have attempted to strip words of their semantics by contrasting homophonous pseudowords in noun and verb context (to wug vs. the wug), thus providing a tool for ascertaining differential representation of lexical categories (Cappelletti, Fregni, Shapiro, Pascual-Leone, & Caramazza, 2008; Laiacona & Caramazza, 2004; Shapiro & Caramazza, 2003; Shapiro, Moo, & Caramazza 2006; Shapiro, Pascual-Leone, Mottaghy, Gangitano, & Caramazza 2001). However, taking on the role of an *advocatus diaboli*, one might still argue that the phrase “to wug” suggests an action (e.g., whacking) whereas the context “the wug” is more compatible with an object (a rug) interpretation and, therefore, these pseudowords were not truly stripped of semantic associations, but were, in fact, semantically biased by the contexts in which they were presented: as the authors did not explore this possibility empirically, this interpretation (which has earlier been suggested and supported by Pulvermüller, Kherif, Hauk, Mohr, and Nimmo-Smith (2009) and Vigliocco et al. (2011)) cannot be ruled out at this point. Further evidence for representation of lexical categories in the brain comes from differential brain activity in response to homophonous noun and verb affixes presented in noun and verb contexts (Pulvermüller & Shtyrov, 2009), which persist even after the contributions of the noun/verb stems are subtracted. Differential neural responses to acoustically identical affixes of nouns and verbs would appear to strongly support genuine differentiation between lexical categories at the inflectional level. However, since these affixes most typically appear in the context of action verbs and object nouns, respectively, it is entirely probable that their neuronal circuits bind with the semantic knowledge attached to their companion words. Even such surprising noun/verb distinctions in brain activation patterns may, therefore, be traced to a semantic origin.

Indeed, whilst the bulk of evidence regarding noun and verb processing fails to replicate clear brain activation differences between these lexical categories and is frequently confounded by semantics (see Vigliocco et al. 2011, for review), there is unambiguous evidence that semantic associations alone, when disentangled from and unconfounded by lexical category differences, differentially activate cortical areas. This has been concisely addressed by the exploration of different semantic categories within the same lexical class. Action words (verbs) semantically related to the different effectors of the body have been robustly shown to produce differential somatotopic activity in motor systems (Aziz-Zadeh & Damasio, 2008; Boulenger, Hauk, & Pulvermüller 2009; Cappa and Pulvermüller, 2012; Hauk, Johnsrude, & Pulvermüller 2004; Hauk, Shtyrov, & Pulvermüller 2008; Kemmerer, Castillo, Talavage, Patterson, & Wiley, 2008; Pulvermüller, Härle, & Hummel 2001), and likewise, nouns with strong gustatory, olfactory or auditory associations have been shown to differentially activate these respective sensory brain regions (Barrós-Loscertales et al. 2012; González et al., 2006; Kiefer, Sim, Herrnberger, Grothe, & Hoenig 2008). These sensorimotor activations specific to the semantic category of linguistic symbols (words) occur in conjunction with left-perisylvian area activations generally seen during language processing. These semantic activation topographies support a model of language processing based on Hebbian cell assemblies that bind together distributed semantic category-specific sensorimotor and left-hemispheric perisylvian language circuits (Pulvermüller, 1999; Pulvermüller, 2002; Pulvermüller, 2012; Pulvermüller, 2013). The functional relevance of sensorimotor activation for language processing has been demonstrated by causal effects of sensorimotor cortex activation on the processing of specific semantic types of symbols (Boulenger et al. 2006; Devlin & Watkins, 2007; Glenberg & Kaschak, 2002; Glenberg, Sato, & Cattaneo 2008a; Pulvermüller, Hauk, Nikulin, & Ilmoniemi 2005) and by a range of patient studies (Bak, O’Donovan, Xuereb, Boniface, & Hodges, 2001; Pulvermüller et al. 2010; for discussion, see Kemmerer et al., 2012). It therefore appears that differences in meaning between linguistic symbols are manifest in neuronal circuits with specific brain topographies.

Whilst neural differentiation between semantic categories is relatively well-supported, the influence of lexical categories in modulating brain activity is, for the previously mentioned reasons, still undetermined. The optimal design for comparing lexical (noun/verb) and semantic (action/object relationship) differences in brain activation would vary these factors independently. Earlier work explored this strategy using EEG (Brown & Lehmann, 1979; Kellenbach, Wijers, Hovius, Mulder, & Mulder, 2002; Pulvermüller, Mohr, & Schleichert, 1999) and fMRI (Vigliocco et al. 2006) but, especially in the fMRI studies, it was not always possible to control all relevant confounds in an optimal fashion. For example, Vigliocco et al. (2006) compared Italian nouns and verbs with sensory or motor features and found a semantic-topographical but not a lexical class difference. However, a shortcoming of this study was that their Italian noun/verb stimuli shared stems but differed in their affixes (e.g. noun “arrivo” [-O] and verb “arrivare” [-ARE]) and no stimulus matching for word length, word frequency or other lexical variables was reported. This study, as many earlier ones, did not exclude important psycholinguistic confounds which might have led differences in brain activation between nouns and verbs to be overlooked. On the other hand, the fact that ”sensory words were judged as less familiar, acquired later, and less imageable than motor words” (Vigliocco et al., 2006, p. 1791) leaves it open whether the observed differences in brain activation between word types were due to their sensorimotor semantics or to other psycholinguistic features. It is therefore of the essence to properly address the issue of putative lexical–grammatical class differences in brain activation with these pitfalls avoided, and in particular to examine the relationship of lexical class differences to the semantic differences in brain activation reported by the aforementioned authors.

The debate concerning lexical vs. semantic differences as the primary factor for neural differentiation might be addressed with the exploration of well-matched word categories orthogonalised for semantic and lexical factors, such that the contribution of these factors to brain activation in specific cortical areas can be clarified. Whilst nouns and verbs have generally been investigated in the context of concrete items which refer respectively to objects and actions in the world (e.g. “door” and “speak”), they are also highly typical as abstract items generally used to speak about abstract concepts or feelings (e.g. “despair” and “suffer”, “idea” and “think”) and therefore possessing few, if any, sensorimotor associations. Using typical nouns and verbs of a concrete or abstract semantic nature, we here tested predictions of theories of lexical and semantic category representation in the human brain. The lexical–grammatical approach to category-specific local brain processes postulates that the differences in word-elicited cortical activation landscapes are best described in terms of the lexical (or grammatical) categories of nouns and verbs (Daniele et al. 1994; Miceli, Silveri, Nocentini, & Caramazza, 1988; Miceli et al., 1984; Shapiro, Shelton, & Caramazza 2000; Shapiro et al. 2001; Caramazza and Shelton, 1998; Bedny, Caramazza, Pascual-Leone, & Saxe, 2012; Bedny et al. 2008; Laiacona & Caramazza, 2004; Shapiro & Caramazza, 2003). This position implies that the same differences are present for concrete and abstract members of these lexical categories.

In contrast, a semantic approach postulates a difference in brain activation topographies only for concrete nouns and verbs semantically related to objects and actions respectively, but not for abstract nouns and verbs, which lack such clear differences in semantic links with action and perception information. The grounded semantics position views semantic representations as circuits tying together symbolic word form information with action and perception schemas (Barsalou, 1999; Lakoff, 1987). In this perspective, neuronal circuits in motor systems (the neural substrate of action schemas) contribute to semantic knowledge about action-related verbs, whereas meaning knowledge related to object words, typically concrete nouns, is underpinned by neuronal assemblies reaching into inferior-temporal cortex of the ventral-visual “what” stream of object processing (Barsalou, 2008; Gallese & Lakoff, 2005; Martin, 2007; Pulvermüller, 1999; Pulvermüller & Fadiga, 2010). Cortical areas associated with movement or object perception, in middle temporal and inferior temporal/fusiform gyrus respectively, may house additional perceptual schemas related to actions and objects. Abstract words which belong to the noun and verb categories, but which cannot be differentiated from each other based on action- or perception-related semantic features, are hypothesised to evoke similar topographical patterns of brain activation. Previous studies of abstract language processing have implicated a wide range of brain regions, including multimodal dorsolateral prefrontal (Binder, Westbury, McKiernan, Possing, & Medler 2005; Boulenger et al., 2009; Moseley, Carota, Hauk, Mohr, & Pulvermüller 2012), anterior temporal (Patterson, Nestor, & Rogers 2007) and superior parietal cortex (Binder et al. 2005). As a number of studies on abstract word processing have previously found activation in premotor and prefrontal cortex (Moseley et al., 2012; Pexman, Hargreaves, Edwards, Henry, & Goodyear, 2007; Pulvermüller & Hauk, 2006), it seems to be reasonable to predict such activation for our present abstract items, without any further prediction about differences between abstract nouns and verbs.

With tight matching of stimuli and the use of event-related functional resonance imaging (fMRI), we here address the debate around the question as to whether brain activation topographies elicited by words are driven by lexical or semantic factors, or by both. In an orthogonalised design, we presented participants with concrete nouns, concrete verbs, abstract nouns and abstract verbs, hypothesising that differential brain activation between concrete items but not between abstract ones would support a view of semantics driving brain differences between nouns and verbs (reflected in an interaction effect of lexical category and abstractness). Divisions between nouns and verbs but *not* between abstract and concrete items of the same lexical category, reflected in a main effect of lexical category, would imply that the differential topographies for nouns and verbs are driven by the grammatical categories that these items belong to, rather than their varying semantic associations.

## 2 Materials and methods

### 2.1 Subjects

Participants (*n* = 18) were right-handed, monolingual native speakers of English all of whom had no history of psychiatric or neurological illness and were free of psychotropic medication. They had normal or corrected-to-normal vision as suitable for a task within the visual modality. The mean age of participants was 29 (SE = 2.8), all were strongly right-handed (mean laterality quotient = 90, SE = 3.1, Oldfield, 1971), and the group had a mean IQ slightly above average (mean = 110, SE = 3.0) as tested using Form A of the Cattell Culture Fair test (Cattell & Cattell, 1960). Ethical approval was obtained from the Cambridge Psychology Research Ethics Committee (CPREC 2008.64): after receiving written and verbal briefing concerning the full nature of the experiment, participants gave written consent and were all remunerated for their time.

### 2.2 Stimuli

In order to disentangle the effects of lexical category from semantic-abstractness, four word categories of 40 words each were employed (please see Appendix A). Abstract nouns (such as ‘clue’, ‘jape’, ‘truce’) were contrasted with concrete nouns (‘mouse’, ‘cheese’, ‘spade’), abstract verbs (‘faze’, ‘bide’, ‘glean’) and concrete verbs (‘peel’, ‘chomp’, ‘skate’). Prior to the fMRI study, 10 native speakers of English were recruited to provide ratings for a large word corpus on a range of semantic variables. These covered aspects of sensorimotor features, such as imageability, concreteness, visual-relatedness, form-relatedness, colour-relatedness and action-relatedness, and affective-emotional features such as arousal and valence (Bradley & Lang, 1994; Osgood, Suci, & Tannenhaus 1975). Details of the behavioural procedures are described elsewhere (Pulvermüller, Lutzenberger, et al., 1999a, Pulvermüller, Mohr, et al., 1999b). The psycholinguistic properties of words were obtained from the CELEX database (Baayen, Piepenbrock, & van Rijn, 1993), and stimulus groups were consequently matched on length, bigram and trigram frequency, logarithmic lemma frequency, and number of orthographic neighbours (see Appendix B for psycholinguistic and statistical properties of the stimuli). Our study included lexically unambiguous nouns and verbs; lexically ambiguous noun/verbs (such as “the/to walk”) were allowed if their lemma frequencies indicated a dominant usage as either verb or noun. Statistically, the noun lemma frequencies of items in the noun word category by far outnumbered their verb lemma frequencies (abstract nouns: *t*(39) = 4.574, *p* < .000 l; concrete nouns: *t*(39) = 7.891, *p* < .0001), and the reverse was true for the verbs (concrete verbs: *t*(39) = −10.950, *p* < .0001; abstract verbs: *t*(39) = −24.240, *p* < .0001). As can be seen in Appendix B, there were no main effects (or indeed interactions) of lexical category or semantic-abstractness on psycholinguistic properties of stimuli. This being the case, we were confident that brain activation in contrasts focusing on lexical category and semantic-abstractness were free of ulterior confounding effects.

The experimental word categories were dispersed among 200 filler words during presentation, with which they were matched in length (*F*(1, 359) = 1.006, *p* > .436), bigram (*F*(1, 359) = 1.679, *p* > .084) and trigram frequency (*F*(1, 359) = .868, *p* > .560). 120 hash marks, matched to word stimuli in length, acted as a low level visual baseline in contrasts.

### 2.3 Procedure and experimental design

Adopting a paradigm previously employed for investigating lexicosemantic processing (e.g., Hauk et al. 2004; for review, see Pulvermüller et al. 2009), words written in lowercase letters were presented tachistoscopically while haemodynamic responses were recorded using event-related fMRI. This passive reading paradigm was chosen to be unbiased towards semantic or grammatical processing. Despite no overt instructions for semantic processing, it is reliably known to evoke early differential activations that reflect a word’s semantic category (see Hauk et al., 2008, for review), strongly implying that reading automatically evokes semantic processing of word stimuli in typical participants. Subjects were instructed to attend to and carefully read all stimulus words silently, without moving their lips or tongue. The passive reading task was delivered in three blocks of approximately 7 min each. A short presentation time of 150 ms ensured that saccades were discouraged and that participants had to continuously attend to the screen in order to perform the task. A central fixation cross was displayed between stimuli for an average 2350 ms, with a jitter of ±250 ms, resulting in SOAs between 2250 and 2750 ms (average 2500 ms). The order of stimuli was pseudo-randomised (restriction: not more than two items of the same category in direct succession) with two lists, counter-balanced across subjects.

Following the scan, our participants were requested to complete a short unheralded word recognition test outside the scanner. In the recognition test, they were presented with a list of experimental stimuli and novel words and had to rate each word on a scale from 1 to 7, indicating how certain they were that a given item had appeared in the fMRI experiment. For evaluation, ratings were converted into percentage correct/incorrect responses. The test contained a combination of 50 experimental and 25 novel distracter words, and above chance performance was thus taken to confirm that subjects had engaged with the task.

### 2.4 Imaging methods and data analysis

A Siemens 3T Tim Trio (Siemens, Erlangen, Germany) with a head coil attached was employed during data collection. The functional scans consisted of 32 slices which covered the whole brain in descending order (slice thickness: 3 mm, in-plane resolution: 3 × 3 mm, inter-slice distance: 0.75 mm), and echo-planar sequence parameters were TR = 2000 ms TE = 30 ms and flip angle = 78 degrees.

SPM5 (Wellcome Department of Imaging Neuroscience, London, UK) was employed for all processing stages. Images were corrected for slice timing and re-aligned to the first image using sinc interpolation. The EPI images were co-registered to the structural T1 images, which were normalised to the 152-subject T1 template of the Montreal Neurological Institute (MNI), and the resulting transformation parameters applied to the co-registered EPI images. During this pre-processing, images were resampled with a spatial resolution of 2 × 2 × 2 mm and spatially smoothed with an 8-mm full-width half-maximum Gaussian kernel. Single-subject and second level statistical contrasts were computed using the canonical Haemodynamic Response Function (HRF) of the general linear model, a measure for the amplitude of brain response. Low-frequency noise was removed by applying a high-pass filter of 128s. Onset times for each stimulus were extracted from Eprime output files and integrated into a model for each block in which each stimulus group was modelled as a separate event. Group data were then analysed with a random-effects analysis. Activation to each of the experimental word categories was compared statistically against baseline (the hash mark condition) and subsequently between critical stimulus conditions (nouns vs. verbs and abstract vs. concrete words, see below). Stereotaxic coordinates for voxels are reported in the Montreal Neurological Institute (MNI) standard space.

In addition to whole brain analysis, a regions of interest (ROI) analysis was undertaken in which 2 mm-radius regions were defined using the MarsBar function of SPM5 (Brett, Anton, Valabregue, & Poline 2002). This analysis employed both an apriori (theory-led) and a data-driven approach. In the former, a number of coordinates were identified and taken from previous literature concerning category-specific effects for concrete objects in frontotemporal cortex (Chao, Haxby, & Martin 1999; Martin & Chao, 2001; Martin & Weisberg, 2003; Martin, Wiggs, Ungerleider, & Haxby, 1996). Regions were also examined from the recent work of Bedny et al. (2008), who used a motor localiser to identify areas activated by biological motion (left and right area MT+, left and right superior temporal sulcus respectively) and a semantic decision task to identify areas activated by the contrast of action verbs vs. animal nouns (left tempero-parietal junction, left and right anterior superior temporal sulcus).

In a similar fashion, in our data-driven approach, we extracted the regions where clearest evidence for activation (in terms of error probabilities/*t*-values) was found in the contrast of all experimental words pooled together against the baseline, plotted at an FDR-corrected significance level of *p* < .05. The criteria for ROI selection was based on *t*/*Z*-values-obtained in this contrast: we selected the three “most significant voxels”, i.e. those with the highest *t*/*Z* values for the words vs. baseline contrast. As this comparison (words vs. baseline) is orthogonal to both of the variables investigated (lexical category, abstractness), the strategy applied for selecting ROIs follows recent recommendations to avoid “double dipping” (Kriegeskorte, Simmons, Bellgowan & Baker, 2009). In this data-driven analysis, average activation values within each of these 2 mm-radius spheres for each subject and each of the four word categories were entered into a repeated-measures ANOVA with the factors ROI x lexical category (2) × semantics/abstractness (2).

Note that, because 2 × 2 × 2 mm voxels, 8 mm smoothing kernel and 2 mm ROI radius were chosen, the half maximum width of each ROI was 12 mm. This allowed us to keep overlap between ROIs to a minimum while at the same time compensating for some of the spatial variance caused by the projection of individual brains to the averaged MNI template. Where appropriate, Huynh–Feldt correction was applied to correct for sphericity violations. In this case, epsilon values and corrected *p* values are reported throughout.

## 3 Results

### 3.1 Behavioural results

Whereas psycholinguistic properties were matched between word groups (see Methods, Appendix B), results of the semantic rating study executed prior to the fMRI experiment revealed significant differences in the semantic variables of imageability, arousal, action-relatedness, concreteness, visual-relatedness, colour-relatedness and form-relatedness (see Appendix B). For all of these features, 2-way ANOVAs revealed significant interaction effects and, in most cases, additional main effects. The interactions of all object-related features, including concreteness, imageability, form- and visual-relatedness, showed, as expected, highest values for concrete nouns towering over all other word groups. For arousal and action-relatedness, which both reflect semantic action features, concrete verbs achieved the highest ratings and concrete nouns the lowest. In addition, object-related semantic ratings were higher for nouns than for verbs and higher for concrete items than for abstract ones; with regard to action-relatedness, verbs dominated over nouns and, again, concrete over abstract items.

Statistical tests for word groups, including interactions and main effects, are displayed in Appendix B. Pairwise comparisons between stimulus groups showed that the abstract noun category was indeed significantly less imageable (*t*(78) = −14.028, *p* < .001), less concrete (*t*(78) = −16.812, *p* < .001), less related to visual objects (*t*(78) = −15.145, *p* < .001), and less form/shape-related (*t*(78) = −10.443, *p* < .001) than concrete nouns. Likewise, abstract verbs were significantly less imageable (*t*(78) = −8.613, *p* < .001), less concrete (*t*(78), and less action-related (*t*(78) = −3.018, *p* < .005) than concrete verbs. There were no significant differences between the two abstract categories in imageability (*t*(78) = .809, *p* > .421), visual- (*t*(78) = 1.364, *p* > .175) or form-relatedness (*t*(78) = 8.54, *p* > .395), though abstract verbs were significantly less concrete than abstract nouns (*t*(78) = 2.206, *p* < .031). As expected, the most highly imageable category, concrete nouns, significantly outperformed concrete verbs in imageability (*t*(78) = 8.988, *p* < .001), concreteness (*t*(78) = 18.307, *p* < .001), and visual- (*t*(78) = 9.814, *p* < .001) and form-relatedness (*t*(78) = 4.861, *p* < .001).

On the surprise word recognition test performed after scanning, performance was above chance (average hit rate: 76.2% (SE = 4.2%), false positive rate: 56.8% (SE = 5.2%), d’prime rate: 0.53). Although these results only document moderate recognition of stimulus words, possibly due to the large number of the stimuli presented and the long delay between experiment and later testing outside the scanner (∼23 min average), they document that subjects had been attentive during passive reading. In order to check that concrete items were not processed any more thoroughly than abstract ones, d’prime values were calculated for each word category. The average d’primes for each category were as follows: concrete nouns = .024; concrete verbs = 0.59; abstract nouns = 0.52; abstract verbs = 0.56. One-sample *t*-tests revealed that the d’prime of each word category was significantly above 0 (concrete nouns: *t*[17] = 2.092, *p* < .05; concrete verbs: *t*[17] = 4.135, *p* < .002; abstract nouns: *t*[17] = 3.324, *p* < .005; abstract verbs: *t*[17] = 3.669, *p* < .003). A two-way ANOVA (lexical category × concreteness) revealed no significant main effects or interactions between the d’primes of different word categories, such that there was no behavioural evidence for processing differences between word categories.

### 3.2 fMRI results

**Fig. 1:**
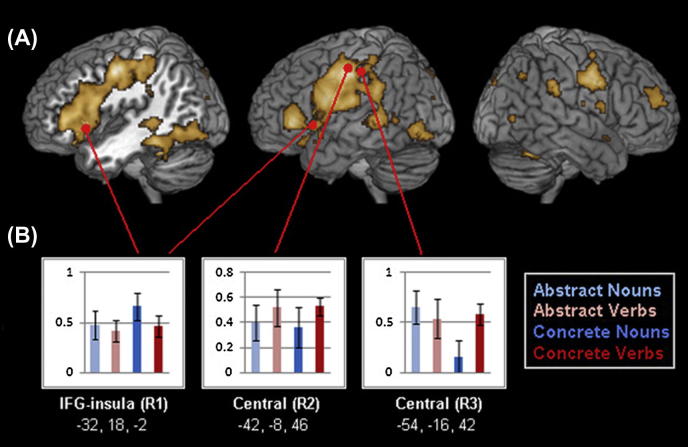
Brain activation elicited by concrete and abstract nouns and verbs. (A) Activation overlays evoked by all experimental words against baseline (hashmarks), plotted at *p* < .05 (FDR-corrected). (B) Activation evoked by each word group in the three ROIs defined around the most highly significant maxima for this contrast. ROIs are located in inferior frontal gyrus (IFG) and underlying insula, in precentral cortex and in central areas.

Examination of the contrast of all experimental words against the hashmark baseline, presented at an FDR-corrected significance level of *p* < .05 in Fig. 1 part A, revealed activation typical of that generally seen in visual language processing tasks (Bookheimer, 2002; Kronbichler et al. 2004). A very large left-hemispheric cluster extended from inferior frontal gyrus (pars orbitalis (BA 47), pars triangularis (BA 45) and pars opercularis (BA 44)) over precentral and postcentral gyrus to supramarginal gyrus, down over superior, middle and inferior temporal and fusiform gyrus, and even back to inferior occipital cortex. Other left-hemispheric clusters included the middle cingulate, parietal and superior occipital cortex and the cerebellum. Activation was also observed in the right hemisphere, with large clusters located at right middle frontal cortex, precentral gyrus and the right cerebellum (close to fusiform gyrus), and a smaller cluster at right supramarginal gyrus. Activation maxima for this contrast are displayed in Appendix C.

### 3.3 Data-driven ROI analyses

Using a data-driven approach, we examined stimulus-induced activation dynamics in ROIs where clearest word-related activation was present. In the contrast of all words vs. baseline, three left frontocentral activation clusters stood out with regard to their low *p*- and high *t*-values (*t* > 6.5; see Fig. 1, and Appendix C) (see also Methods). Activation evoked by the four word categories at these three foci, located in inferior frontal cortex and insula (−32, 18, −2), on the precentral gyrus (−42, −8, 46) and across the central sulcus (−54, −16, 42), was entered into a 3 (ROI: inferior frontal, precentral, central) by 2 (Lexical category: noun/verb) by 2 (Semantics: concrete/abstract) ANOVA. Crucially, a significant interaction of all three factors, ROI, Lexical category and Semantics (*F*(2, 34) = 4.002, *p* < .028), demonstrated that the four word categories evoked significantly different topographic activation patterns across these three frontocentral regions. (Fig. 1B).

To further investigate this complex interaction, separate analyses of variance were carried out for concrete and abstract words (design: ROI × Lexical category [nouns vs. verbs]). For concrete nouns and verbs, there was a significant interaction of the ROI factor with Lexical category (*F*(2, 34) = 4.38, *p* < 0.020). Planned comparison tests failed to reveal a category difference in the inferior frontal and precentral ROIs, but documented stronger haemodynamic activity in central motor cortex for concrete action-related verbs than for object-related nouns (*F*(1, 17) = 5.66, *p* < 0.029) and a tendency in the opposite direction for the inferior frontal ROI (*F*(1, 17) = 2.227, *p* > .15). When grouping together premotor and motor ROIs (i.e. precentral and central), significantly stronger responses to concrete verbs than to concrete nouns were re-confirmed (*F*(1, 17) = 5.74, *p* < 0.028). The same two-way analysis of variance carried out for abstract nouns and verbs failed to reveal a significance interaction effect of the ROI and Lexical category factors (*F*(2,34) = 0.79, *p* > 0.46, n.s.). There was no indication of word category differences in motor, premotor or prefrontal areas of interest. This pattern of results shows that only concrete action-/object-related nouns and verbs, but not abstract ones, activate the frontocentral areas differentially.

Further inspection of activation patterns to abstract and concrete nouns and verbs in the three ROIs suggested that, over and above the statistically confirmed category-difference for concrete but not abstract items, the abstract items seemed to group with action verbs. Pooling haemodynamic responses to abstract words with those to concrete action verbs did indeed confirm significantly greater activity in the central motor ROI than that evoked by concrete nouns (*t* [17] = 2.285, *p* < .04). The precentral region indicated the same trend but without reaching significance. The inferior frontal ROI showed a trend towards stronger responses to concrete nouns compared with the other three categories, though this did not reach significance (*t*(17) = 1.351, *p* > .195).

### 3.4 A-priori defined ROI analyses

**Fig. 2:**
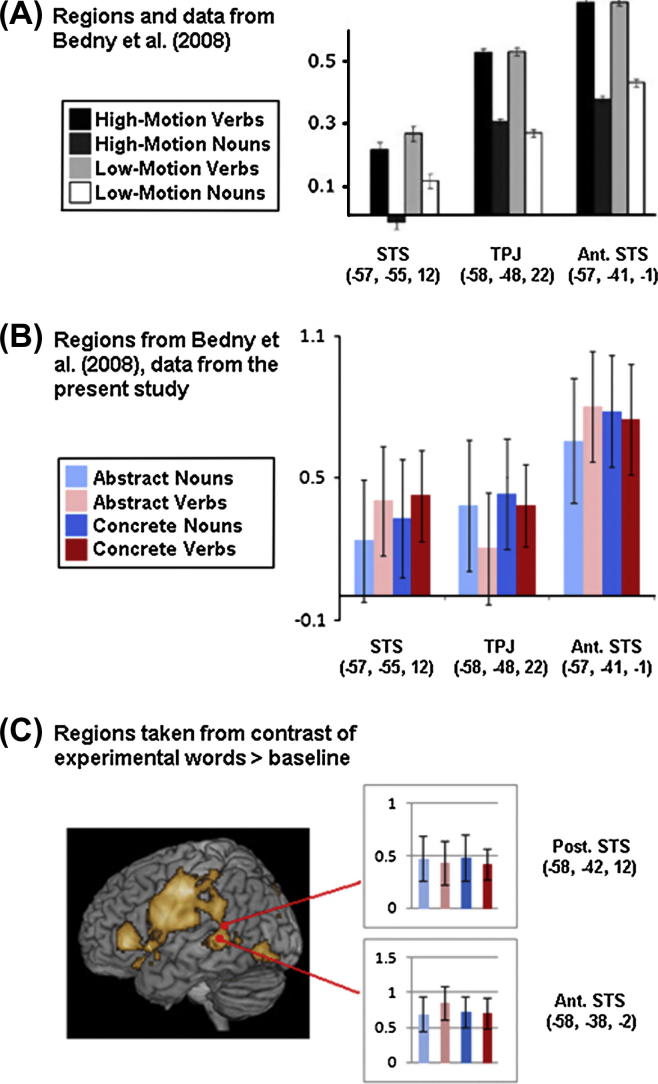
Left-temporal brain activations elicited by noun and verb categories. Comparison between earlier reports and the present results. (A) ROIs and data from Bedny et al. (2008): averaged relative signal change (in arbitrary units) during word comprehension vs. baseline. High- and low-motion nouns and verbs are depicted in shades of grey in these three ROIs (superior temporal sulcus [STS], temperoparietal junction [TPJ], and anterior superior temporal sulcus [aSTS]). (B) Activity evoked in the same regions by abstract and concrete nouns and verbs in the present study. (C) Activation to abstract and concrete nouns and verbs in regions around local activation maxima in the contrast of all words vs. baseline. In this case, we selected local activation maxima in posterior and anterior STS which were close to the activation foci shown in A.

In a second set of analyses, we investigated the word-type related activation in regions highlighted in previous research. ROIs were therefore taken from the literature and effects of lexical category or semantics were investigated by two-way ANOVAs. Previous work targeting both lexical category differences (noun–verb) and semantic dissociations (living–nonliving, animals–tools, etc.) was exploited in defining ROIs (Bedny et al. 2008; Chao et al. 1999; Martin & Chao, 2001; Martin & Weisberg, 2003; see Vigliocco et al. 2011). Bedny et al. (2008) reported a significant effect of lexical category but NOT of the only semantic variable they focused on, motion—related semantic word properties. This lexical category effect was seen in three ROIs, where verbs evoked greater activity than nouns: left temperoparietal junction (TPJ: coordinates −58, −48, 22), superior temporal sulcus (STS: −57, −55, 12) and anterior superior temporal sulcus (aSTS: −57, −41, −1). However, using the same ROIs to scrutinise the present data set, we could not observe any concordant significant effect, and, more generally, not any main effect or interaction of either Lexical category or Semantics (all *F*-values <0.5). Their left STS ROI revealed a trend towards a lexical category difference which, though weak and far below significance (*F*(1, 17) = .422, *p* > .525), somewhat resembled that reported by Bedny, with numerically greater activity for verbs (see Fig. 2, Part A). No significant effect of lexical category appeared in either the left temperoparietal junction ROI (*F*(1, 17) = .400, *p* > .536) or the left anterior superior temporal sulcus ROI (*F*(1, 17) = .105, *p* > .750); in these cases, any numerical differences in favour of verbs were also absent in our data, in favour of a numerical contrast in the opposite direction. The combination of all three Bedny et al. regions (TPJ, STS and aSTS) also failed to reveal a significant effect of lexical category or semantics. Although, in our present analysis, activation maxima did not arise in the left STS in the contrast of all experimental words against baseline, we chose two coordinates located in the cluster of STS activation which were closest to Bedny et al.’s original anterior and posterior STS regions (see Fig. 2B). These coordinates, too, failed to replicate the verb advantage reported by Bedny and colleagues in left STS and showed no effect of lexical category. The present study was therefore unable to replicate the noun/verb difference in haemodynamic responses previously reported in left middle-temporal cortex.

**Fig. 3:**
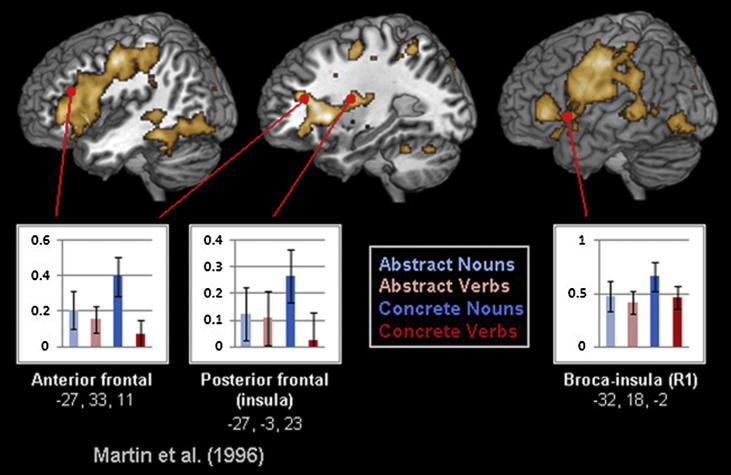
Analysis of activity to concrete and abstract nouns and verbs in inferior frontal ROIs taken from Martin et al. (1996). The observed activation strength for concrete nouns in inferior frontal regions shows similarity with the pattern obtained from the IFG-insula region of the data-driven analysis (see Fig. 1).

So far, analysis of all ROIs from the previous literature failed to reveal effects of lexical category. We did, however, observe a main effect of lexical category in analysis of two left frontal-insula regions (one more anterior at MNI coordinates −27, 33, 11, the other more posterior at −27, −3, 23) suggested by Martin et al.’s (1996) results of a positron emission tomography (PET) experiment investigating the naming of visually depicted animals and tools (*F*(1, 17) = 6.280, *p* < .025). Of more important note, though, was the significant interaction between this lexical category factor and the semantics variable (*F*(1, 17) = 9.319, *p* = .007). This interaction was driven by significantly greater activation for concrete nouns (see Fig. 3) compared with concrete verbs in both the more anterior first (*t*(17) = 2.301, *p* < .035) and posterior (*t*(17) = 3.046, *p* < .01) frontal regions. Whilst nouns generally evoked greater average activation than verbs in these regions, the difference between abstract nouns and verbs did not reach significance in the present study. Comparison of brain responses to concrete nouns to the pooled response to all three other word types confirmed the relatively enhanced signal to the former in the anterior ROI (*t*(17) = 2.611, *p* = .018) and a trend in this direction in the posterior (*t*(17) = 1.672, *p* = .113). Note, furthermore, the similarity between the activation advantage for concrete nouns in this ROI defined by Martin et al. (1996) and the data-driven IFG/insular ROI found in the present study. Martin et al. had investigated animal and tool naming and these ROIs showed strongest responses in animal naming; in our study, which used words in a passive reading task, most of the concrete nouns were also animal names. The inferior frontal region thus appears particularly engaged in animal name processing, regardless whether this occurs during naming or passive reading.

## 4 Discussion

In a study of abstract and concrete noun and verb processing, we found a significant interaction effect of orthogonalized semantic (abstract vs. concrete) and lexical (noun vs. verb) factors in the frontocentral motor system. In central and precentral motor cortex, activation to concrete verbs was generally enhanced compared with concrete nouns and, crucially, a similar difference for abstract word groups was absent. Inferior frontal regions suggested the opposite contrast, activation greater for concrete nouns than for concrete verbs, but, once again, the contrast of nouns vs. verbs was not significant for abstract items. As statistically significant effects of lexical category appeared in interaction with semantic differences between abstract and concrete words, our results argue against a distinction between topographical patterns of brain activation in terms of the lexical categories of nouns and verbs. Rather, our data show that brain activation patterns to nouns and verbs depend on the semantic nature of these items. The most prominent brain distinctions include enhanced activity in central motor cortex to verbs typically used to speak about actions relative to object-related nouns, and relatively stronger activation in inferior frontal cortex to object nouns as compared with action verbs.

Our neurometabolic data reveals a pattern of activation across frontal and temporal cortices typical of that generally seen in visual word processing (Bookheimer, 2002; Kronbichler et al., 2004). It is also consistent with the aforementioned model of distributed word-related cell assemblies, showing activation spread out across left perisylvian language cortex and incorporating semantic circuits in extrasylvian regions, especially motor and visual areas (Pulvermüller, 1999; Pulvermüller, 2002; Pulvermüller, 2012; Pulvermüller, 2013). In an investigation of three frontal regions (in IFG-insula, precentral and central gyrus) most significantly active during processing of experimental words, a region (3) by semantic abstractness (2) by lexical category (2) ANOVA revealed a significant interaction of all three factors. Further investigation confirmed the lexical category difference in brain activation patterns for concrete but *not* for abstract items. These results show that noun/verb differences in brain activation patterns are specific to concrete items and therefore depend on semantics.

A search for effects of lexical category in temporal regions implicated in previous literature was unfruitful, though a lexical category effect did appear in two frontal regions previously implicated by Martin et al. (1996) in the processing of animal pictures. This effect was driven by a particular strength for concrete nouns, which were indeed mainly animal words, as consistent with this and other previous studies reporting substantial activation overlap in this area for animal concepts across modalities (Martin, 2007; Martin & Chao, 2001). Considering the theoretical models previously discussed, our findings demonstrate greater support for a semantic than a lexical interpretation of focal neurometabolic noun/verb differences, but demand a more complex discussion of the impact of lexical category and semantics on the brain.

### 4.1 Lexical categories in the brain

The proposition that lexical (grammatical) categories are differentially represented in the brain would seem plausible given that nouns and verbs are suggested by many to be linguistic universals (Vigliocco et al., 2011), even present in American Sign Language (ASL: Supalla and Newport, 1978), pidgin and creole languages (Slobin, 1975). Exceptions do exist (Broschart, 1997; Foley, 1998; Langacker, 1987; Robins, 1952), however, such that linguists now query whether these categories are truly shared cross-culturally across languages (Croft, 2001; Kemmerer & Eggleston, 2010). Nouns and verbs are defined combinatorially and due to the extreme diversity of language systems (some which lack inflectional categories and function word types, for example), it is clear that the combinatorial criteria for inclusion in the noun/verb categories must differ between languages. At present, the brain-imaging work on nouns and verbs assume that these categories are valid in the Western population (speakers of English or European languages such as Italian and German) and that, therefore, it is possible that these categories have a shared and specific basis in the brain. It is this claim that we investigate here; the wider notion that all language-speaking individuals have inborn brain representational systems for these grammatical categories cannot be ascertained, as it is uncertain whether these conceptual categories can be applied to all languages in the same manner. The semantic feature that words are used to speak about actions or objects seems to be shared by many, if not all, languages and therefore would provide a solid basis for a cross-linguistic distinction.

Based on previous evidence from neuropsychological, neurophysiological and neurometabolic investigation, a range of authors have suggested that the lexical/grammatical category of words might be the primary dimension by which neural segregation is driven (Shapiro et al. 2000; Shapiro et al. 2001; Caramazza and Shelton, 1998; Bedny et al. 2008; Cappelletti et al., 2008; Laiacona & Caramazza, 2004; Mahon & Caramazza, 2008; Shapiro et al., 2006; but see also Damasio & Tranel, 1993; Daniele et al., 1994; Gainotti, 2000; Luzzatti et al. 2002). This idea is founded on noun and verb dissociations in patient studies (Bak, O’Donovan, Xuereb, Boniface, & Hodges 2001; Bak et al. 2006; Boulenger et al., 2008; Cappa et al., 1998; Cotelli et al. 2006; Damasio et al. 2001; Daniele et al. 1994; Miceli et al., 1984; Miceli et al., 1988; Shapiro & Caramazza, 2003), electrophysiological studies (Brown, Lehmann, & Marsh 1980; Dehaene, 1995; Preissl, Pulvermüller, Lutzenberger, & Birbaumer 1995; Pulvermüller, Lutzenberger et al., 1999; Pulvermüller, Mohr et al., 1999; Pulvermüller, Preissl, Lutzenberger, & Birbaumer 1996) and metabolic imaging studies (Perani et al. 1999; Warburton et al., 1996). As such, some authors, such as Bedny et al. (2012), suggest that language processing and conceptual representation is amodal and functionally separate from perceptual and action systems of the brain. This view has a rich tradition in approaches to cognitive science (Anderson, 2003; Fodor, 1985; Machery, 2007), viewing the manipulation of abstract amodal symbols as a core component of mental functions. The amodal symbolic system would interface with sensorimotor systems only for receiving its input or passing on its output, but otherwise maintain functional separation from those brain systems concerned with action and perception (cf., for example, Bedny et al., 2012; Mahon & Caramazza, 2008; Pylyshyn, 1984). Therefore, this position interprets the noun/verb dissociations found in clinical and neurofunctional studies in the sense of a lexical category difference unrelated to semantics.

Problematically, as mentioned in the introduction, nouns and verbs differ on a range of dimensions uncontrolled for in many of the studies mentioned in the previous paragraph. These features are either semantic in nature (as many nouns relate to objects whereas most verbs are used to speak about actions) or immanent to psycholinguistics measures (for example word frequency) or more general linguistic features (for example to the degree to which combinatorial grammatical information is linked to classes of lexical items) (see, for example, Bird, Howard, & Franklin 2001; Bird, Lambon Ralph, Patterson, & Hodges 2000; Gainotti, 2000; Pulvermüller, Lutzenberger et al. 1999). Therefore, although noun/verb dissociations in patient populations and differential brain activation to these categories have been reported in the studies above, it is unclear to what degree such dissociation depends on linguistic and semantic features of these word groups.

In an attempt to take these confounds into consideration, Bedny et al. (2008) focused on nouns and verbs varying in semantic features, especially in their semantic relationship to motion perception. We would like to consider these findings in detail as, despite a similar design, Bedny and colleagues’ stimulus selection along with their results dramatically differ from those reported here. Contrary to previous studies (Martin et al. 1996), these authors reported that activity in middle temporal regions close to motion-sensitive areas “responded preferentially to verbs relative to nouns, even when the nouns have higher visual-motion properties” (than verbs) (p. 11352) and hence suggested that “concepts… are organised according to conceptual (lexical) properties” (p. 11347). In their attempt to tease apart lexical and semantic factors, these authors controlled semantic aspects related to visually perceived motion, grouping together animal nouns and action verbs as “high motion” items in spite of their fundamental differences with regard to a range of semantic dimensions. This neglect and lack of control for semantic aspects of verb and noun stimuli is a major shortcoming, as previous work has documented brain activation differences related to semantic action- vs. object-relatedness, manipulability of referent objects of nouns, or action-relatedness of verbs (see next section; Brambati et al. 2006; Damasio et al. 2001; Martin & Weisberg, 2003; Pulvermüller et al. 2009; Tranel, Martin, Damasio, Grabowski, & Hichwa 2005). Bedny’s comparison of “high-motion” noun and verb categories, namely animal names and action verbs (such as “sheep” vs. ”grasp”), is problematic, as we have demonstrated in previous work that many animal words lack action-semantic links and, correspondingly, fail to elicit action-related brain activity, whereas action verbs, which represent the prototype of action-related lexical materials, activate cortical motor systems along with middle-temporal cortex (Moseley et al., 2012). It has indeed been suggested that the middle-temporal activation might reflect visual motion processing, but there is so far no firm proof for this hypothesis and general action-relatedness provides at least one alternative cognitive-semantic feature that may be reflected (Kiefer et al. 2012). Because likely semantic determinants of their middle-temporal activations were not sufficiently documented, the noun/verb difference in brain activation observed by Bedny et al. *cannot* be seen as unrelated to semantics.

With greater control of semantic stimulus properties related to action and perception, our present findings as summarised in Fig. 3 indeed failed to support the hypothesis brought forward by Bedny et al. that noun/verb differences might be sufficient for differential middle-temporal activation. This was true in spite of the care taken to replicate the exact regions of interest where Bedny and colleagues found their effects, and we even explored adjacent regions where activation maxima were observed in our present data set. Any significant main effects of lexical class were absent both in Bedny et al.’s left STS and temperoparietal ROIs and in adjacent ROIs defined in a data-driven manner. Although there was a weak tendency in the previously reported STS ROI towards higher activity for verbs, the opposite trend emerged from both TPJ and aSTS regions. Therefore the present data fail to confirm the conclusions drawn by Bedny et al. A recent review concludes that, after exclusion of linguistic and semantic confounds, any possible differences between the grammatical categories of nouns and verbs are weak if present at all (Vigliocco et al. 2011). Our work leads us to concur that there is, to date, no unambiguous evidence for lexical category differences in middle temporal cortex. More generally, our present results seem to discourage the idea that lexical differences per se are reflected at brain-level by different areas for either “nouns” or “verbs”.

### 4.2 Semantic categories in the brain

Whilst our findings belie local dissociation between words on the sole basis of lexical category, they are consistent with a semantic approach postulating that the *meaning* of words is reflected in differential brain activation topographies elicited when these words are recognised and understood. Any topographical difference in brain activation to concrete nouns and verbs, or neuropsychological dissociations between the same, would, accordingly, be a consequence of the fact that these items are typically used to speak about objects and actions respectively (Gainotti, 2000; Pulvermüller & Fadiga, 2010; Pulvermüller, Lutzenberger et al. 1999; Pulvermüller, Mohr et al. 1999; Shallice, 1988).

The modulation of frontocentral brain activity by semantic features of stimulus words in the present study, especially the stronger activation seen in the central motor region to concrete action verbs compared with concrete object nouns, is consistent with a wealth of literature showing semantically-driven differences in word-elicited brain activation (Aziz-Zadeh and Damasio, 2008; Barrós-Loscertales et al., 2012; Boulenger et al., 2009. Gainotti, 2000; González et al., 2006; Hauk et al., 2004; Kemmerer et al., 2008; Kiefer et al., 2008; Pulvermüller et al. 2001; Tettamanti et al. 2005; Willems, Hagoort, & Casasanto 2010). The appearance of dissociations *within* grammatical categories, for example between face-, arm- and leg-related verbs (Hauk et al., 2004) and between action- and sound-related nouns (Kiefer et al., 2012; Trumpp, Traub, & Kiefer, 2013) is strong evidence for semantic modulation of neural response to words which is independent of lexical category.

Patient studies, too, would appear to support this interpretation. Deficits for processing tool concepts and words are associated with frontoparietal sensorimotor systems (Gainotti, 2004; Gainotti, Silveri, Daniel, & Giustolisi 1995) and deficits for animals with occipitotemporal regions (Hart & Gordon, 1992; Tranel, Damasio, & Damasio 1997). These dissociations appear to be underpinned by the dissociation between action- and perception-related knowledge, with manipulability and other action-features most relevant for tools, and visual-features such as colour and form most relevant for animals. More recent work with stringent psycholinguistic matching has revealed relative impairments for action-word processing in a range of neurological diseases and disorders characterised by motor impairment (Bak et al., 2001; Bak et al. 2006; Boulenger et al., 2008; Cappa et al., 1998; Cotelli et al., 2006; Moseley et al., 2013). Importantly, deficits in processing action language, associated with lesions to inferior frontal and motor systems, are accompanied by concordant deficits in semantic processing of actions in *nonverbal* tasks (Bak et al. 2006). This pattern of deficits provides further evidence for a semantic rather than grammatical basis of category-specific semantic and conceptual disorders, a position reached by two recent reviews of the literature (Kemmerer et al., 2012; Kiefer & Pulvermüller, 2012).

The conclusions drawn in the present paper are consistent with previous works but avoid some of the methodological pitfalls evident in the same. Vigliocco et al. (2006), as in the current paper, reported brain dissociations between sensory and motor words but no distinctions on the basis of lexical category. Problematically, this study used Italian nouns and verbs sharing the same stem but differing in their affixes, which immediately inform the reader of the word’s lexical category. The co-occurrence of verb affixes with verb stems (used to speak about actions) and the co-presence of noun affixes with nouns (related to objects) appears to indirectly load the neuronal circuit of affixes with semantic links (Pulvermüller & Shtyrov, 2009). The study also suffered from poor stimulus matching, such that apparent dissociation between motor and sensory words might also be explained by differences in familiarity, imageability and age of acquisition (see, for example, Hauk et al., 2008). Other electrocortical dissociations on the basis of both lexical *and* semantic distinctions were reported by Kellenbach et al. (2002) and Barber, Kousta, Otten, and Vigliocco (2010). Whilst these could not be localised to specific brain regions in the former, the latter argued that, as both differences showed the same N400 topography, they might both best be explained in terms of a semantic effect (Barber et al., 2010). Whilst we too would support primary dissociations between word types at the semantic level, the N400 can reflect a range of different psycholinguistic features (e.g., Hauk, Davis, Ford, Pulvermüller, & Marslen-Wilson, 2006) so that a strong conclusion on semantics being the only relevant variable required more support from an experiment avoiding major psycholinguistic confounds.

In light of these flaws in pre-existing research, our present study using well-matched stimulus materials, spatially precise event-related fMRI and a fully orthogonal design crossing the effects of lexical category and semantic type now provides strong support that action- and object-related referential semantics but not lexical categories (noun/verb) are reflected at brain-level by a topographical distinction between motor systems and inferior-temporal activations. The current work can therefore corroborate some of the statements made by studies above which, due to their methodological flaws, could not be strongly defended the findings reported here suggest that previously reported noun/verb differences in the brain were driven by semantics. This position seems consistent with an EEG study, where Pulvermüller, Mohr et al. (1999) reported neurophysiological dissociations between action verbs and object nouns, which were closely paralleled by the contrast between action and object nouns, but no evidence for neurophysiological dissociations between action nouns and verbs.

A lack of neurophysiological and neurometabolic differences in brain activation patterns elicited by the lexical categories might lead some to suggest that lexical categories are illusory, lacking a brain basis – an argument that would of course be flawed. Apart from their semantic differences, nouns and verbs are distinct in their combinatorial properties: English nouns combine with articles and adjectives, and verbs combine with nouns, pronouns and specific prepositions or complementizers. It is necessary to neurally represent the different combinatorial properties of these words in the brain, and the imprinting of different combinatorial patterns of nouns and verbs in a neurocomputational model induces fine-grained connection differences at the neuronal circuit level which provide a neuromechanistic correlate of combinatorial lexical categories (Buzsáki, 2010; Pulvermüller, 2010; Pulvermüller & Knoblauch, 2009). However, such differences at the micro-circuit level, related to the combinatorial properties of nouns and verbs, may be too fine-grained to become manifest as differential brain activations revealed by standard neuroimaging techniques (fMRI, EEG or MEG). As such, with the data available at present, these topographical differences between word types are best explained in semantic terms, as outlined in the following section.

### 4.3 Neuronal dissociations between concrete nouns and verbs as explained by the cell assembly model

Differential activation was found for concrete nouns and verbs, whereby the latter activated motor and premotor areas more strongly than the former and the opposite contrast was significant in inferior frontal cortex. These results are consistent with and explained by a neuromechanistic model rooted in Hebb’s concept of distributed cell assemblies. Because knowledge about the form and meaning of a word are normally active together such that neuronal connections between the respective neuronal circuits are strengthened, these meaning- and form-related circuits are joined together into one higher-order semantic network – to the degree that one circuit part typically does not activate without the other becoming active too. There is room for flexibility in this mechanism, especially if attentional resources are limited, overt motor action is being prepared for, or context puts a focus on grammatical processing (Angrilli, Dobel, Rockstroh, Stegagno, & Elbert 2000; Chen, Davis, Pulvermüller, & Hauk 2013; Hoenig, Sim, Bochev, Herrnberger, & Kiefer 2008; Pulvermüller, Cook, & Hauk 2012; Rueschemeyer, Lindemann, Van Elk, & Bekkering 2009; van Elk, van Schie, Zwaan, & Bekkering 2010). However, for typical passive tasks (reading, listening), action-related verbs activate semantic circuits involving motor and action schemas stored in motor and premotor cortex, and a wealth of neuroimaging and neuropsychological work indicates that this activation is functionally important for action word processing (Buccino et al. 2005; D’Ausilio et al. 2009; Devlin & Watkins, 2007; Glenberg & Kaschak, 2002; Moseley et al., 2013; Pulvermüller et al. 2005; Shebani & Pulvermüller, 2011).

For object-related nouns, visual knowledge about objects stored in inferior-temporal areas is of special relevance. Previous research (Kiefer et al., 2008; Kiefer et al., 2012; Martin et al., 1996; Pulvermüller & Hauk, 2006) has documented focal differences between fine-grained word types in temporal cortex. This was not replicated in our dataset, possibly because our concrete noun category lacked semantic uniformity, including nouns from several different semantic categories which may have led to a mix of temporal region activations and weighed against semantic dissociations. For example, the concrete noun category was predominantly dominated by animal names, which were rated as strongly semantically-related to form knowledge (Appendix B)*.* Pre-existing work reported that form-related words activate inferior frontal areas (−46 28 10; Pulvermüller & Hauk, 2006), such that the current activation advantage for concrete nouns in more anterior inferior frontal cortex (−27 33 11) may be hypothesised to reflect form knowledge immanent to animal concepts. In this context, it is important to recall that our inferior frontal ROIs, where concrete nouns activated more strongly than concrete verbs, were motivated by previous work by Martin and colleagues, who reported stronger activation during animal naming compared with tool naming in these regions (Chao et al. 1999; Martin & Chao, 2001; Martin et al. 1996). However, as other concrete nouns were also part of this lexico-semantic subcategory, it is not surprising that any inferior-frontal effect potentially related to form-semantics did not yield clear significant results.

### 4.4 Abstract nouns and verbs and the motor system

It may come as a surprise that abstract nouns and verbs activated the motor systems to a similar degree as action verbs. There are however theoretical arguments for involvement of motor systems in abstract meaning processing. For abstract words typically used to speak about emotions and internal states of the body, semantic theory postulates that these are learnt when word form and state-/emotion-expressing actions are linked with each other (Baker & Hacker, 2009; Wittgenstein, 1953) – a prediction consistent with motor activity evoked by emotion-related words (Moseley et al. 2012). (Note that abstract emotion words may be both nouns and verbs (e.g. (the) fear), and, therefore, a degree of motor activation to the nouns and verbs in this study can be explained). Abstract metaphors, idioms and other types of abstract concept, including numbers, have also been suggested to be intrinsically linked with visually-observable behaviours and actions (Boulenger, Shtyrov, & Pulvermüller 2012; Boulenger et al., 2009; Glenberg et al. 2008b; Tschentscher, Hauk, Fischer, & Pulvermüller 2012) or arrangements/relationships in space (Casasanto, 2009; Lakoff & Johnson, 1980) that represent typical instantiations of their abstract meaning. In this view, knowledge about actions and perceptions and corresponding processes in sensorimotor areas of cortex play a role in abstract concept and meaning processing (Barsalou, 1999; Gallese & Lakoff, 2005; Kiefer and Pulvermüller, 2012; Lakoff & Núñez, 2000; Wilson-Mendenhall, Barrett, Simmons, & Barsalou, 2011).

Abstract nouns and verbs can, of course, differ semantically both between *and* within their lexical categories, and in order to obtain a representative sample of abstract items from each lexical category, it was not possible to focus on specific semantic subclasses of abstract terms in this present work. Our results are therefore consistent with a fundamental role of motor systems in abstract word and concept processing, as suggested above. On theoretical grounds, the cell assembly model predicts comparably weak sensorimotor links for some abstract terms (e.g., “beauty” and “justice”), because their semantic manifestation in action and perception is quite variable and therefore correlation learning predicts relatively weak links between sign and concept. We did not find a general difference in activation between our strongly action-related verbs and the abstract categories here, but, as mentioned, this may be due to the stimulus selection, especially a low proportion of abstract terms with variable semantics in the present stimulus set. In this context, it is noteworthy that Pexman et al. (2007) also found sensorimotor activation for both abstract and concrete concepts but in their study activation to the former was weaker than that to the latter, which is consistent with somewhat weaker sensorimotor semantic links in cell assemblies for abstract semantics.

## 5 Conclusions

This study investigated whether topographical patterns of brain activation to nouns and verbs are driven by lexical (grammatical) class or rather by semantics and word meaning. We found that concrete nouns and verbs activate frontocentral cortex to different degrees. Whereas motor and premotor areas are relatively more strongly activated by action verbs, concrete nouns activated more anterior prefrontal areas. At the cognitive level, these differential activations appear to relate to the processing of action schemas that are part of the semantic representation of action verbs and of form knowledge semantically linked to object words. Abstract nouns and verbs fail to elicit similar activation differences, thus calling into question previous claims about genuine brain loci for the major lexical categories. Systematic investigation of other areas, especially temporal cortex, also failed to reveal a genuine distinction between noun and verb processing loci. We suggest that topographical brain activation differences elicited by words are driven by semantic factors and that the lexical category distinction is mechanistically implemented at a level beyond the grain size of neurometabolic imaging.

## Funding

This work was supported by the MRC (MC_US_A060_0034, U1055.04.003.00001.01 to F.P., MRC studentship to R.M.), EPSRC and BBSRC (BABEL grant), DFG (Center of Excellence “Languages of Emotion”) and Freie Universität Berlin.

## Figures and Tables

**Table:** 

Abstract nouns	Abstract verbs	Concrete nouns	Concrete verbs
Dole	Skimp	Mouse	Bash
Clue	Glut	Shark	Slash
Wares	Bilk	Cow	Jive
Speck	Stash	Trout	Pat
Loot	Coax	Flute	Swig
Graph	Snare	Torch	Delve
Tune	Heal	Spade	Steer
Luck	Cite	Skunk	Knit
Chore	Glean	Crab	Chat
Knell	Parse	Seal	Chomp
Pledge	Soothe	Crow	Hike
Draft	Dwell	Cream	Knead
Fluke	Pall	Duck	Peel
Mote	Fend	Cress	Skate
Yield	Fetch	Worm	Drone
Farce	Waive	Beet	Snort
Jape	Faze	Axe	Chew
Hint	Soak	Dove	Braid
Bloom	Lapse	Sloth	Skim
Guide	Drift	Hawk	Stride
Fleck	Slake	Snake	Poke
Dream	Trust	Hare	Hack
Truce	Shirk	Harp	Croon
Scope	Feign	Goose	Sprint
Lump	Lure	Cheese	Munch
Goal	Stun	Kale	Carve
Truth	Shine	Soup	Slink
Batch	Botch	Goat	Wade
Wealth	Strive	Frog	Lisp
Aid	Bid	Flan	Choke
Flow	Dare	Deer	Bind
Fraud	Quell	Rice	Grope
Gale	Bate	Pig	Scrape
Score	Cease	Cake	Tug
Theme	Lodge	Sheep	Strut
Crime	Tempt	Bull	Pinch
Term	Fade	Bran	Scuff
Spur	Reap	Whale	Frisk
Realm	Taint	Shrimp	Stab
Lint	Bide	Toad	Slur

**Table:** 

	Abstract nouns	Abstract verbs	Concrete nouns	Concrete verbs
Length	4.63 (.10)	4.6 (.01)	4.38 (.01)	4.58 (.01)
Bigram freq.	40794.5 (3344.74)	41159.19 (2868.66)	39189.07 (2745.79)	36984.03 (2099.55)
Trigram freq.	4166.72 (1025.41)	4503.48 (732.12)	4293.83 (429.39)	3529.272 (419.95)
Log. lemma freq.	1.26 (.10)	1.26 (.11)	1.18 (.08)	1.35 (.07)
No. of neighbours	6.38 (.86)	6.4 (.86)	8.28 (.90)	6.53 (.86)

Imageability	2.49 (.20)	2.28 (.16)	6.05 (.16)	4.13 (.14)
Concreteness	3.06 (.17)	2.61 (.11)	6.42 (.10)	3.53 (.12)
Visual-relatedness	2.31 (.21)	1.97 (.14)	6.02 (.13)	3.72 (.20)
Form-relatedness	1.50 (.13)	1.37 (.09)	3.37 (.13)	2.38 (.16)
Action-relatedness	2.58 (.22)	4.16 (.20)	1.76 (.15)	4.91 (.14)
Arousal	1.71 (.11)	2.04 (.16)	1.43 (.11)	2.98 (.15)
Valence	3.89 (.20)	3.64 (.17)	3.81 (.08)	3.56 (.15)
				

**Table:** 

	Main effect of lexical category (*F*)	Main effect of semantic-abstractness (*F*)	Interaction: lexical category × semantic-abstractness (*F*)

Length	.681 (*p* > .410)	1.682 (*p* > .197)	1.126 (*p* > .290)
Bigram freq.	.108 (*p* > .743)	1.065 (*p* > .304)	.211 (*p* > .647)
Trigram freq.	.094 (*p* > .760)	.368 (*p* > .545)	.623 (*p* > .431)
Log. lemma freq.	.960 (*p* > .329)	.001 (*p* > .981)	.938 (*p* > .334)
No. of neighbours	.980 (*p* > .324)	1.350 (*p* > .247)	1.038 (*p* > .310)
Imageability	40.841 (*p* < .001)	264.807 (*p* < .001)	26.524 (*p* < .001)
Concreteness	167.848 (*p* < .001)	276.123 (*p* < .001)	89.628 (*p* < .001)
Visual-relatedness	58.773 (*p* < .001)	251.677 (*p* < .001)	32.085 (*p* < .001)
Form-relatedness	19.342 (*p* < .001)	128.860 (*p* < .001)	11.379 (*p* < .001)
Action-relatedness Arousal	170.307 (*p* < .001)	.036 (*p* < .850)	18.553 (*p* < .001)
Valence	48.320 (*p* < .001)	5.921 (*p* < .016)	20.437 (*p* < .001)
	2.505 (*p* < .116)	.278 (*p* < .599)	.000 (*p* < .992)

**Table:** 

	*x*	*y*	*z*	*Cluster size*	*t*	*P*
*Experimental words*						
L. insula (bordering on BA 47)	**−32**	**18**	**−2**	8522	6.90	<.001
L. central gyrus (BA 4)	**−54**	**−16**	**42**		6.67	<.001
L. precentral (BA 6)	**−42**	**−8**	**46**		6.54	<.001

R. middle cingulate (BA 24)	10	12	26	987	6.47	<.001
(cluster extending rightwards from cingulate, touching on the caudate)	22	10	28		5.47	<.001
	28	14	20		5.41	<.001

R. precentral gyrus (BA 6)	60	2	36	429	5.91	<.001
R. precentral gyrus (BA 6)	58	−2	44		5.39	<.001

L. superior occipital cortex (BA 19)	−12	−84	42	761	5.56	<.001
L. cuneus (BA 18)	−2	−84	26		4.55	<.001
R. cuneus (BA 19)	10	−82	22		4.07	<.001

L. middle cingulate (BA 32)	−8	20	38	974	5.30	<.001
	−12	4	34		5.05	<.001
L. SMA (BA 6)	−2	0	60		4.58	<.001

R. cerebellum crus. 1/fusiform	36	−54	−32	562	5.22	<.001
R. cerebellum 6 (BA 19)	26	−64	−24		5.09	<.001
R. cerebellar vermis 6	6	−72	−18		3.86	<.001

R. middle frontal cortex (BA 46)	30	44	16	519	4.77	<.001
R. middle frontal cortex (BA 46)	36	56	22		4.09	<.001
R. middle frontal cortex (BA 46)	42	46	28		3.37	<.002

R. supramarginal gyrus (BA 40)	66	−36	34	100	4.61	<.001

Deep parietal	−16	−46	46	137	4.15	<.001
L. superior parietal (BA 7)	−22	−56	56		3.67	<.001

Very bottom of BA 30	−14	−32	−32	39	4.02	<.001

L. cerebellum	−10	−54	−18	42	3.73	<.001

L. cerebellar vermis/lingual cortex	−4	−62	0	197	3.71	<.001
L. lingual cortex (BA 17)	−2	−72	0		3.68	<.001
L. cerebellar vermis	−2	−54	−6		3.26	<.002

R. cerebellum	6	−56	−20	190	3.65	<.001
R. cerebellum (BA 18)	10	−56	−6		3.58	<.001

L. cerebellum (BA 19)	−32	−66	−26	70	3.60	<.001

## Appendix A

Experimental word stimuli (presented in lowercase).

## Appendix B

Psycholinguistic and semantic properties of word stimuli. Statistical analysis of variance (ANOVA) between word groups is displayed in *F* values, as are possible interactions and main effects.

## Appendix C

MNI coordinates evoked by the contrast of all experimental words against a low-level visual baseline (###), calculated at an FDR-corrected significance level of *p* < .05.The maxima from the most highly significant cluster, which were the focus of our analysis, are highlighted in bold.
